# Using Ultra-Abridged Individual Difference Scales for Personalization in Digital Mental Health to Improve Uptake, Engagement, and Experiences: Three-Tiered Decision Framework for Scale Shortening

**DOI:** 10.2196/80662

**Published:** 2026-06-08

**Authors:** Siu Kit Yeung, Alan C Y Tong, Han Zhao, Winnie W S Mak

**Affiliations:** 1 Department of Psychology, Chinese University of Hong Kong, Rm 333, Chen Kou Bun Building, Hong Kong, China (Hong Kong), 852 3943 6577

**Keywords:** personalization, scale shortening, digital mental health, individual differences, uptake, engagement

## Abstract

Given the diversity of human characteristics and experiences, personalization in nudges, messages, choice presentations, interventions, and overall product design has been increasingly adopted in digital health to promote engagement. Past studies on moderators and personalization in digital health and mental health services generally focused on demographic and symptom variables, with generally inconsistent findings or null findings. Cognitive, motivational, and decisional psychological attributes are largely overlooked. Psychology often uses long self-report scales to measure various psychological attributes. Although they are useful in tapping into individuals’ psychological profiles, when applied in real-life, everyday settings to assess individual differences, people are most likely unwilling to complete them. With the pressing need to personalize digital health platforms to enhance uptake, retention, and engagement, ultrashort versions of these psychological scales may be considered to allow assessment of multiple attributes at the same time. Scale shortening can be achieved through regression analyses of each item, factor analyses, item response theory, ant colony optimization, and machine learning methods, with each method having advantages, disadvantages, and conditions required to make it suitable. To illustrate, we provided examples of regression analyses of each item and factor analyses, with potential implications for personalizing narrative versus research-based messages in digital mental health contexts. We present a 3-tiered decision framework for scale shortening method selection depending on goals and possible constraints, with guidelines on validation methods for ultrashort scales. Moving forward, more validation studies and field studies in digital health platforms are needed to evaluate the ecological validity, reliability, and generalizability of these methods, bearing in mind the limitations and conditions where such shortening methods may not work well. Researchers may compare the effectiveness and limitations of personalization using ultrashort scales with other commonly adopted personalization methods (eg, based on longer scales, behavioral data, and large language models). Ethical concerns need to be considered and mitigated carefully, respecting diverse preferences, informed choices, and the privacy of service users. Our viewpoint piece is primarily intended for digital mental health researchers and practitioners, but may also be informative for the fields of digital health and medicine as well as personalization (eg, personalized health care, personalized nudging, and message matching) more broadly, given the common goal of boosting uptake and engagement as well as improving service users’ experiences.

## Scale Length as a Barrier to Personalization in Digital Mental Health

Human beings are heterogeneous, with diverse personalities, social-cognitive characteristics, decision-making tendencies, motivational orientations, perceptions, and experiences, etc [1]. Given that one size does not fit all, personalizing messages, choice presentations, nudges, user interface designs, and interventions are generally more effective in encouraging uptake of healthy behaviors, promoting engagement and health outcomes [1-12]. Such generally positive effects are supported or partially supported by numerous reviews, including meta-analyses in various contexts, including digital health environments [2,8,10-13]. Evidence of personalization advantage in digital mental health contexts is more mixed, with some studies showing the advantage of personalized services over nonpersonalized services [14,15]. However, there are studies demonstrating positive effects of personalized digital mental health services overall but not detecting differences between interventions with and without personalization components (see the recent reviews by Dandil and Kingston [14] and Schaeuffele et al [15]). Pinpointing the causes of such discrepancies is premature, since null findings do not imply the absence of advantage and digital mental health studies comparing personalized conditions with nonpersonalized conditions are limited [14-16].

Past studies regarding personalization or moderation of mental health services have generally focused on demographic and disorder-focused variables (ie, depression symptom severity, anxiety severity, and clinical diagnosis [17,18]). However, the effects of personalizing based on these variables are inconsistent [17,18], and some service users are concerned about privacy issues with sharing such potentially sensitive mental health information [19,20]. Personalization based on motivation, decision tendencies, and cognitive styles may be promising as they have demonstrated positive effects in the broader health behavior and tailoring or matching literature (see reviews by Joyal-Desmarais et al [2] and Nikoloudakis et al [13]). These psychological attributes capture the variability of humans in ways that are inherently humanistic rather than medical. Many of these variables are closely associated with health decisions [9] and thus provide digital systems with valuable information to respond to the whole person in more nuanced and effective ways. With the availability of numerous validated individual-level psychological scales, measuring them for personalization of digital health services is potentially feasible [21,22]. One major challenge is the length of these scales, especially when added together. To tap into multiple psychological characteristics, users may need to complete dozens or even hundreds of items. Yet, most users are not willing to complete such long questionnaires [23]. Relatedly, many users of digital mental health platforms may be experiencing mental health challenges, which may affect their cognitive load and motivation [24]. Even though users may look for personalized care, completing long questionnaires may require substantial cognitive and motivational resources that add a burden to these already-distressed individuals, possibly leading to abandonment of the platform or service altogether [24,25].

## Ultrabrief Scales in Digital Mental Health

To tackle this practical obstacle, we propose shortening psychological scales significantly to an ultrabrief version (ie, 2‐4 items [13,14,16]), with empirical steps to retain acceptable reliability and validity, so that personalization can be made feasible without overburdening users [15]. In this viewpoint piece, we aim to (1) introduce and recommend various scale shortening methods for personalization purposes in digital health with examples (refer to “Item Selection and Scale Shortening” as well as “Selecting Items for Personalization” sections), (2) discuss validity, reliability, and ethical considerations (refer to “Validity and Reliability Considerations” as well as “Ethical Considerations” sections), and (3) propose a decision framework that takes account into purposes, execution considerations, and constraints while maintaining validity and reliability as well as examining real-world applicability and effects (refer to “Recommendations on Practical Framework” section). We hope this piece will drive more research studies on ultrashort scales for personalization, facilitating applicability and improving user experiences in personalization. We consider digital mental health researchers and practitioners as the primary audience of this piece, as personalization is a popular engagement-boosting method in digital mental health spaces [16]. Given widespread implementations of personalization [4], this piece is also potentially relevant for digital health researchers and practitioners in other non–mental health domains as well as researchers and practitioners who are interested in personalization of health-related services, recommendations, messages, choice presentations, and nudges.

For decades, the use of very short scales to measure health-related constructs has been widely adopted in public health contexts. While there are many studies on scale shortening in the broader psychology and health-medical literature, work validating or applying brief scales for personalization in digital mental health contexts remains scarce. One exemplar is the shortening of Patient Health Questionnaire-9 (PHQ-9) [26] and Generalized Anxiety Disorder-7 (GAD-7) [27] into Patient Health Questionnaire-4 (PHQ-4) [28] that incorporated 2 items from each [29,30]. Hlynsson et al [30] found that Patient Health Questionnaire-2 (PHQ-2) and Generalized Anxiety Disorder-2 (GAD-2) are effective at identifying people with significant symptoms (with satisfactory discriminative validity) throughout different stages, including pretreatment, midtreatment, and posttreatment phases. When compared to their full versions, the short forms demonstrated good reliability and validity, especially at posttreatment for GAD-2. The PHQ-4 has now been used in digital platforms and mobile apps as a screening measure and assists in content, service, or platform recommendations or personalizations [31,32]. Given that the anxiety and depressive symptoms are highly prevalent in the general population, the utility of such short scales can enable quick screening. If non–symptom-focused psychological measurements can also be abridged with sufficient reliability and validity, they can offer a vast array of individual attributes for personalization.

## Item Selection and Scale Shortening

Currently, factor analyses (FAs), item response theory (IRT), ant colony optimization (ACO), and machine learning (ML)–based methods [33-35] are established ways to shorten scales. FA is commonly adopted for scale shortening and may involve selecting items based on higher factor loadings [36,37] and tends to require smaller sample sizes than other methods. The method using IRT selects items based on more detailed information, including measurement precision, item information, and item discrimination, and can maintain precise estimation of the score of the original scale, but requires a larger sample size than FAs [38-40]. ACO selects the superior solution among multiple possible solutions but performs better with 500 or more participants in general [41]. ML methods, notably genetic algorithms, also select the short form with better prediction performances after considering many combinations, but also need larger sample sizes, ideally thousands of participants [33]. Each method has its pros, cons, and practical challenges, which are summarized in Table 1. Researchers may choose the appropriate statistical methods based on the sample size, number of factors, and technical knowledge or experience with relevant methods.

**Table 1.** Explanations, advantages, disadvantages or barriers, and suggested software or programs, packages, and/or modules of different scale shortening methods^a^.

Methods	Explanation	Advantages	Disadvantages or barriers	Suggested software or programs, packages, and libraries
Factor analyses	Selecting items with the highest factor loadings, as shown in EFAs^b^ [36,37].	Relatively feasible and easy for most researchers to implement while not requiring very large sample sizes, with many programs and packages available. Similar goodness-of-fit indices to more complex methods such as ACO^c^ when there is one factor [41].	When there are 2 factors and there is minor model misspecification, poor model-data fit can occur, and FAs^d^ perform worse than ACO [41].	SPSS is a program that requires payment or an institutional subscription, with both point-and--click and syntax functions. The basic SPSS program can run EFA [42], whereas running CFA^e^ requires the Amos add-on [43]. Jamovi [44] is an open-source program that allows point-and-click and syntax, with EFA and CFA functions. R (an open-source program) Package *lavaan* provides more options (such as estimation methods and multigroup CFA) for conducting EFAs and CFAs, with more information reported (such as about goodness of fit) [45]. R Package *psych* can help conduct EFAs and internal consistency tests, with more options available [46].
Item response theory	Selection of items based on detailed information, including measurement precision, item information, and item discrimination of each item [38].	Provides more detailed information for each item, but not simply a single reliability score for the test in FAs [38]. Ensures sufficient measurement precision across the whole range of the trait [38].	More mathematically challenging, and many researchers may not have the relevant fundamentals for implementing IRT^f^. Requires a large sample size, generally 500 participants and ideally larger, which may not be feasible for some researchers and in some contexts given resource constraints [47]	IRTPRO is a stand-alone program designed specifically for various IRT tests, for both unidimensional and multidimensional models with both dichotomous and polytomous data [48]. MPlus is a program that requires entering syntax, with the advantage of integrating IRT with other data analytical methods such as factor analytical methods, mixture modeling and multilevel mixed modeling [49]. R Package *mirt* was signed for multidimensional IRT, with both confirmatory and exploratory modeling [50]. R Package *TAM* is particularly suitable for running IRT tests for large-scale datasets, including multidimensional, multilevel, and multigroup modeling [51,52]. R Package *ltm* is designed for unidimensional IRT but not multidimensional IRT, and may be more suitable for less complex models [53,54].
Ant colony optimization	Involves metaheuristic algorithms to select a better solution among multiple possible solutions, with the goal of maximizing model fit [41,55]	Can better select items with higher data-model fit by considering multiple variations and selecting the superior solution [55]. Outperforms FAs in goodness of fit when there are 2 factors and model misspecification is present [41].	Technically more challenging to implement, especially for researchers without computing fundamentals. Very limited options available that are specifically designed for ACO in short-form development (R Package *ShortForm*). Generally requires a large sample of at least 500 participants, with the sample split into training and validation samples [40]. Such a large sample size may be less implementable in some contexts.	R Package *ShortForm* can facilitate short form creation with the goal of maximizing validity with criteria decided by the researcher [35]. HeuristicLab is a standalone program with ACO functions, requiring users to create fitness or validity criteria for item selection [56].
Machine learning–based methods	Selection of items aiming to predict participants’ sum score or mean score in the original scale, with both training dataset and cross-validation dataset [33]. Considers many possible combinations of short forms in selecting the short form with better prediction performances, through methods such as genetic algorithm [33].	Can better maintain predictive validity [57]. Can predict participants’ scores in the original scale through a small number of items accurately [33].	To achieve high prediction performance, large sample sizes, ideally thousands of participants, are needed [33]. Such sample sizes may not be feasible for some studies. Lack of consistency in items selected in bootstrapped samples [33]. Requires higher proficiency and expertise in machine learning and programming, which is not common in the field of psychology.	R Package *GA* offers general-purpose functions for genetic algorithms, but requires more procedures (such as creating functions) by users to shorten the scales and may be more suitable for users with higher expertise in R [58]. Python DEAP (Distributed Evolutionary Algorithms in Python) is a library in Python that consists of evolutionary algorithms functions, including genetic algorithms, which can be used for optimizing items to be selected [59]. HeuristicLab allows customization of genetic algorithms, which can be used for scale shortening [56].
Item-level moderated regression analyses	Conducting separate regression analyses with interaction between each item of the scale and the condition, possibly with alpha correction. Item selection may be based on 2‐4 items with the largest interaction regression coefficients.	Technically simple to implement. Helps select items with stronger moderation effects, which may be more relevant and efficacious for personalization in a specific context.	The selected items based on (larger) regression coefficients may not capture the entire construct well. The items selected may be sample dependent and may lack generalizability to other samples.	SPSS allows various forms of regression analyses [42], with the Process Macro add-on for simple slope analyses [60]. Jamovi consists of functions for both logistic and linear regression as well as simple slope analyses [44]. R glm and lm functions (base R) can be adopted for logistic regression and linear regression, respectively.

a The above table provides information on explanations, pros and cons, as well as suggested programs, packages, or libraries of different scale shortening methods. This table is not meant to be a comprehensive review of various scale shortening methods but to encourage scale shortening for the purposes of personalization in digital health contexts. The table only provides a very brief summary of various methods. For more details regarding various scale shortening methods, see [33,34,41].

b EFA: exploratory factor analysis.

c ACO: ant colony optimization.

d FA: factor analysis.

e CFA: confirmatory factor analysis.

f IRT: item response theory.

## Selecting Items for Personalization

Researchers may conduct moderation analyses with the abridged questionnaires to examine whether the individual attributes moderate the effects of conditions (eg, messages and therapies) on outcomes such as uptake, engagement, decision, satisfaction, or well-being. If moderation and simple slope effects with the short form are shown to be practically meaningful with cross-over interaction effects (ie, condition A results in better outcomes than condition B for people higher in a psychological attribute whereas condition B leads to better outcomes than condition A for people lower in a psychological attribute, see the example below [61]), further work can examine whether the short form can be applied in a digital platform for personalization. Apart from selecting items based on primary data, researchers are also encouraged to share data openly on repositories such as the Open Science Framework, so that others can conduct secondary analyses to shorten the scales for implementation and/or future studies, perhaps through combining samples from multiple shared datasets, with methods that require larger sample sizes, such as IRT and ML methods [33,38-40].

One overlooked but particularly helpful method for selecting items relevant for personalization is to perform moderation analyses on an item level (that has shown evidence for moderation on a scale level) and select the items (preferably 2 to 4 items) with the strongest moderation effects. This method ensures the items selected work well in personalization in a particular context, while other scale shortening methods may not always achieve this goal. In the following, we will explain with an example of narrative versus research messaging, with moderation of thinking and decision styles on the breathing exercise decision.

In a study of 166 young adults, we compared the effects of narrative versus research evidence messages in encouraging stress management, including playing a breathing exercise video and investigated individual difference moderators while conducting exploratory analyses to shorten scales [61]. Individual difference moderators included Experiential Thinking Style (ETS)—indicating tendencies to focus on past experiences, emotions, and narratives in making judgments and decisions [62], and decision style—categorized as rational decision style (deliberate and systematic tendencies in making decisions) and intuitive decision style (emphasis on feelings and fast judgments in making decisions) [63]. Participants were randomized to the narrative message that involves storytelling of a person implementing stress management methods to cope with distress, or the research evidence message that includes statistical information from research studies (such as systematic reviews) regarding the impact of stress management methods [61]. The logistic regressions with standardized premessage stress management behavior as the covariate showed that ETS [62] and decision style [63] (which were standardized) moderated the effects of narrative versus research evidence messages on breathing exercise practice decision [61].

For people with higher ETS and intuitive decision style, narrative messages resulted in higher likelihood to play the breathing exercise video, perhaps because of the match in information presented (story) and judgment or decision style. In contrast, whereas for people with lower ETS, tentative evidence indicated that research evidence messages may outperform narrative messages [61]. The effect sizes for these differences are medium to large, but with *P* values for the simple slope analyses of .03 and .06 at –1 SD level of ETS and intuitive decision style, respectively [61]. As this is an experimental study with young adults, such evidence is considered preliminary and field studies with more diverse samples and perhaps cross-validations with larger sample sizes will be needed to test the reliability of the findings and to prevent overfitting [33].

Supplementary and exploratory regression analyses found that Item 2 (“I often go by my instincts when deciding on a course of action”) and Item 5 (“I tend to use my heart as a guide for my actions”) in the ETS scale [62] have the largest interaction effects, both with *P* values below adjusted alphas (accounting for multiple exploratory tests). These were assessed using standardized regression coefficients in log-odds metrics and corresponding odds ratios (ORs; refer to Table S1 in Multimedia Appendix 1). Combining these 2 items for further analysis also produced significant results (bs indicate unstandardized regression coefficients of the interaction terms), *b*=1.16, 95% CI 0.46-1.86; OR 3.20, 95% CI 1.59-3.43; *P*=.001. These comparable coefficients suggest that the shortened Experiential Thinking Style captured the same moderation pattern as the full scale (*b*=0.91, 95% CI 0.23-1.60; OR 2.49, 95% CI 1.26-4.93; *P*=.009).

Apart from conventional regression analyses for testing moderation effects, different analytical methods, including multiverse analyses, robustness checks, sensitivity analyses, and ML methods as well as replications may be implemented to compare moderation and personalization effects and/or assess the validity and reliability of findings [33]. If multiple methods show support for sizable moderations of certain terms, such items are more likely to be robust and meaningful moderators.

Results indicated evidence of internal reliability. The internal consistency of the full version was Cronbach *α*=0.67 with the average interitem Pearson correlation as 0.17, and the 2-item short version yielded a Spearman-Brown coefficient of 0.63 with the interitem correlation as 0.46. There was a strong positive correlation between the two versions, *r*(164)=0.84, 95% CI 0.79-0.88; *P*<.001. The short ETS scale also showed positive correlations with the Intuitive Decision Style subscale, *r*(164)=0.74, 95% CI 0.66-0.80; *P*<.001, and negative correlations with the Rational Decision Style subscale, *r*(164)=–0.29, 95% CI –0.42 to –0.14; *P*<.001, providing evidence of convergent validity. Correlations among all scales are presented in Table S5 in Multimedia Appendix 1.

We also conducted similar regression analyses and exploratory factor analyses (EFAs) with Hamilton et al [63] scales, and such results with suggested short forms are reported in Tables S2 and S3 in Multimedia Appendix 1. To summarize, we found support for moderation with both the full version (OR 2.70, 95% CI 1.34-5.45; *P*=.005) and the short form (OR 2.82, 95% CI 1.42-5.61; *P*=.003) of Hamilton et al [63] Intuitive Decision Style. Results indicate evidence of acceptable internal consistency of both long form (*α*=0.88, average interitem correlation=0.54) and short form (Spearman-Brown coefficient=0.88, interitem correlation=0.78). The 2 selected items (Item 6 and Item 8, refer to Multimedia Appendix 1 for the items) have the largest regression coefficients with *P* values below adjusted alphas (Table S3 in Multimedia Appendix 1) and the highest factor loadings based on EFA (Table S6 in Multimedia Appendix 1).

To complement the above regression analyses, we also conducted EFA with both Maximum Likelihood-Varimax and Minimum Residuals-Oblimin, and the results with 2 methods are highly similar. The EFA with 172 individuals in this study revealed a 3-factor solution, with these 2 items (2 and 5) being the highest factor-loading items on 2 factors, respectively (refer to Table S4 in Multimedia Appendix 1 for the values). Although Item 8 (“I generally don’t depend on my feelings to help me make decisions,” reversed, [62]) represents a significant item for the third factor in the EFA, it did not significantly moderate the impact of messages on practice decisions in the item-level regression and is therefore not recommended. Given the goal of item selection is for personalization, an item being significant for a factor does not guarantee its inclusion if the item is not a meaningful moderator. Given these insights, future studies that aim to personalize narrative versus statistical or research evidence messages based on ETS may consider including Item 2 and Item 5 [62] for simplicity to test whether moderation effects exist. Such short forms are intended for testing potential matching and moderation effects in the context of narrative versus statistical or research evidence messages only, but not for other purposes.

## Validity and Reliability Considerations

While ultrashort scales may improve engagement and reduce dropout, they inevitably raise questions about validity and reliability [64,65], which we partially examined in the above examples and will conduct further studies. The key is not to assume equivalence with longer scales but to define acceptable benchmarks and trade-offs given the measurement purpose. For reliability, conventional benchmarks such as Cronbach α will no longer be appropriate given a short scale will almost inevitably yield a lower value of Cronbach α, not necessarily because it is unreliable, but because the formula inherently penalizes brevity [66]. Consider the formula of Cronbach α: *α*=N × *r*/1+(N−1)× *r*, where N is the total number of items and *r* is the mean interitem correlation. For example, a 3-item scale with interitem correlations of 0.50 yields a Cronbach α value of 0.75, while a 10-item scale with the same correlations would reach a Cronbach α value of 0.91. It is not the reliability in itself but the number of items that biases the value downward. Moreover, Cronbach α assumes all items have essentially equal factor loadings (tau-equivalence). This assumption rarely holds in psychological constructs, especially after trimming [67], and violating it causes Cronbach α to misrepresent true reliability [68]. When items differ in their factor loadings, as they often do in ultrashort scales, Cronbach α becomes a particularly inaccurate metric. For 2-item measures, while statistics such as the Spearman-Brown coefficient are recommended as a mathematically superior alternative to Cronbach α [69], these metrics still fundamentally rely on classical test theory assumptions that can become unstable with only 2 data points. Therefore, although they could serve as a reference, traditional reliability tests of this kind are not particularly telling for ultrashort scales.

Instead, we recommend using interitem correlation as the reliability indicator. Briggs and Cheek [70] recommend that the mean of a set of interitem correlations ideally lies between 0.20 and 0.40, whereas greater than 0.50 suggests potential item redundancy. Likewise, Clark and Watson [71,72] recommended that the average interitem correlation and the average interitem correlation for each item fall within the range of 0.15‐0.50. Together, we suggest somewhere around 0.30 (±0.15-0.20) to be acceptable, but these criteria are arbitrary and not universally applicable. Reliability should not be treated as a fixed and sole criterion but interpreted alongside construct coverage and test-retest stability [34,73].

For validity, reducing scales by large narrows conceptual bandwidth, capturing only the most central indicators of a latent construct [74]. To determine whether an ultrashort scale retains construct validity, if feasible, researchers can evaluate both structural equivalence and conceptual correspondence. For structural equivalence, researchers may fit both the full and shortened scales within the same dataset in confirmatory factor analysis if there are 3 or more items, while examining their factor loadings, configural structure, and fit indices [34,41]. If the short scale reproduces the same factor structure with acceptable fit, structural validity is supported [34,41].

For conceptual correspondence, researchers can correlate scores from the short form with the full-scale scores and external theoretically related variables. Convergent correlation over 0.80 or above between the short form and long form may be considered acceptable [75], but there is no well-established acceptable cutoff. When item removal leads to factor distortion or loss of theoretically important variance, further reduction is unjustified [74]. However, confirmatory factor analysis can possibly be done for short forms with 3 items or more, whereas for models with 2 items, it would be just-identified, resulting in a perfect fit. Therefore, in 2-item scenarios, we recommend cross-checking the variance explained with at least 2 datasets and comparing the explanatory variance loss from the shortened scale compared to the full scale (also see the paper by Smith [74]).

## When Using Ultrashort Scales for Personalization Is Problematic

Ultrashort scales should be avoided when measurement precision, reliability, or validity would be compromised [34,65]. They may not be suitable for multifaceted constructs such as mindfulness, where multiple subdomains (cognitive, affective, and behavioral) jointly define the construct [76]. For such constructs, subscales may be more suitable. Constructs that fluctuate rapidly, such as momentary affect and state mindfulness, or other variables that require repeated measurements also cast doubt. Researchers can use experience sampling at different time points using short brief items, preserving representativeness while minimizing fatigue [72].

Ultrashort scales may also be problematic when standardized comparability (eg, a cutoff) is required, as brevity disrupts communication and comparability across research and practice settings [77]. When full instruments are replaced by abbreviated forms, sometimes results may not be well cross-referenced across studies, clinical services, or policy benchmarks, limiting shared understanding and comparability [77]. In such contexts, maintaining fully validated scales ensures consistency, interpretability, and interoperability across systems.

Finally, ultrashort scales risk bias when measurement invariance across demographic or cultural groups is untested, or when the construct itself remains theoretically underdeveloped. In these cases, scale shortening is premature because there is no stable conceptual foundation for deciding which items are essential for content coverage or certain intended purposes such as diagnosis and screening [74]. This occurs when a construct’s definition, dimensionality, or boundaries are still evolving, unclear, or inconsistently operationalized across studies [72]. For example, emerging concepts such as digital alliances are still lacking established theoretical hierarchies. Reducing items without a clear framework risks eliminating important indicators. In such contexts, the priority should be theoretical clarification and factor validation rather than item trimming [72].

## Recommendations on Practical Framework

**Figure 1.**
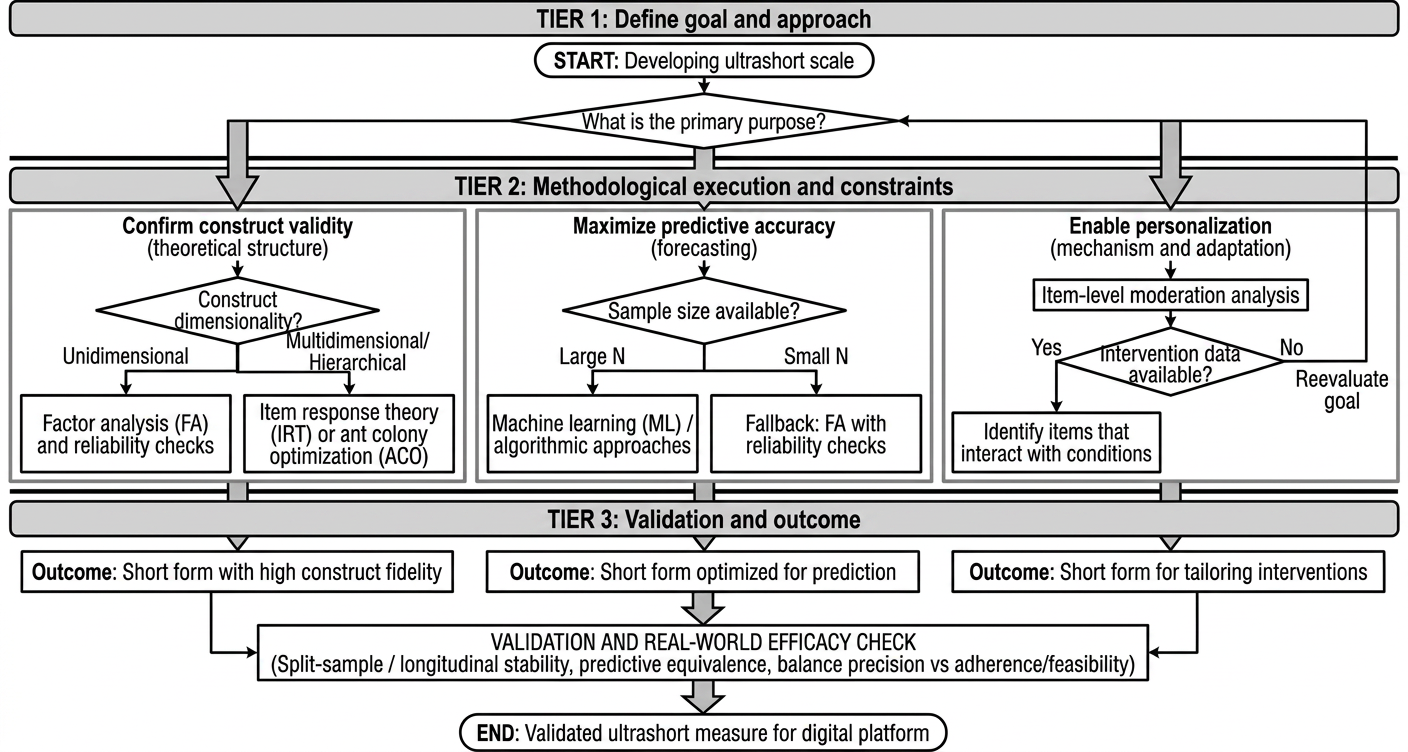
Decision framework. The purposes are not mutually exclusive, meaning there can be multiple purposes in scale shortening. The diagram is created with Gemini (version 3 Pro; Google DeepMind) and Nano Banana Pro (Google DeepMind).

When scale shortening is considered appropriate without substantial problems, researchers developing ultrashort psychological scales for digital platforms can align their reduction methodology with the specific utility of the data. We propose a 3-tiered framework for method selection (Figure 1). For example, a researcher may prioritize both predictive accuracy and identification of items that interact better with conditions. In such situations, a combination of FA or ML approaches plus item-level moderation analyses may be adopted.

### Tier 1: Define Goal

When the goal is to replicate the structure of a full-length assessment (eg, replacing standard intake forms), traditional FA or IRT is recommended [74,78]. These methods prioritize internal consistency and the maintenance of the latent trait.

If the objective is forecasting outcomes, such as behavioral patterns or engagement probabilities, algorithmic approaches such as ML or ACO are generally superior. These methods prioritize variance explanation over theoretical purity [79,80].

When the objective is to personalize, accuracy alone is insufficient. A model may predict engagement with 90% accuracy without revealing how to influence it. While conducting the above scale shortening statistical methods is worthwhile, item-level moderation analyses can be considered. This approach identifies items that interact with intervention conditions to predict differential outcomes. For example, specific items assessing “experiential thinking style” may moderate the effects of narrative versus research-based content. Unlike standard reduction, which discards “noisy” items, personalization retains items that function as distinct mechanisms for adaptation.

The inclusion of a reevaluation loop is critical to safeguard against methodological misalignment. It enforces the sharp distinction between prognostic utility (predicting who is likely to engage or improve) and prescriptive utility (determining which specific intervention will cause that improvement). True personalization relies on detecting heterogeneity of treatment effects, a statistical interaction that mathematically requires variation in intervention conditions [81]. Without distinct data on intervention types (eg, narrative vs didactic), it is premature to isolate the “mechanism of action” required to claim a scale enables personalization. This loop compels researchers to acknowledge this constraint, preventing unsupported claims about adaptability and ensuring they pivot back to methods such as ML or FA that align with the limitations of a static dataset.

### Tier 2: Consider Methodological Constraints

Once the goal is defined, technical constraints dictate the specific analytical execution. We should take into account the factor structure and the sample size available before choosing a suitable method.

#### Factor Structure

The dimensionality of the construct must be revisited prior to item selection. For unidimensional constructs, strong factor loadings and low residual correlations in FA suggest that a few high-performing items can adequately represent the latent trait [82]. However, multidimensional or hierarchical constructs require IRT or metaheuristic methods (eg, ACO) to ensure the short form retains measurement precision across all necessary subdomains without losing content validity.

#### Sample Size Practicality

Method selection is often constrained by data availability. FA can yield stable results with smaller cohorts. In contrast, IRT typically requires n>500 for reliable parameter estimation [83], and ML-based approaches ideally require several thousand participants to minimize overfitting during cross-validation [33]. In data-scarce environments, we recommend generating preliminary short forms via FA and reliability checks to be refined later as sample sizes grow.

### Tier 3: Validation and Real-World Performance

Regardless of the derivation method, rigorous validation is needed. Split-sample or longitudinal validation needs to demonstrate structural stability, acceptable reliability (interitem correlation 0.15-0.50; [72]), and predictive equivalence. Triangulating methods can be considered. For instance, researchers may retain items that possess both high factor loadings and strong IRT information to enhance robustness [84]. Replications with multiple different samples are also recommended.

The predictive performance of ultrashort scales can be compared with the full scale and evaluated in the context of ecological feasibility. While long scales theoretically offer higher measurement precision, their predictive validity often degrades in real-world digital settings due to fatigue and attrition [85]. Well-chosen items often retain distinctively high power. For instance, the PHQ-4 explains 80%‐90% of the variance in depressive and anxiety outcomes relative to the full PHQ-9 and GAD-7, with minimal loss in classification accuracy [28] while providing the benefits mentioned throughout this paper.

## Ethical Considerations

Apart from methodological considerations, researchers and practitioners should consider ethics carefully. Personalization based on a minimal number of items may be preferred by some users but disliked by others, whereas some service users may prefer other methods, such as artificial intelligence chatbot-based personalization but some service users may dislike artificial intelligence–based methods for mental health services [86]. Digital platforms should not impose such methods without the informed consent of users, as this may be perceived as a violation of privacy (see the papers by Matz et al [4] and Teeny et al [8] for related discussions). Transparent, clear, and explicit explanations of personalizations are needed to respect the diverse needs, preferences, and concerns of service users [20]. Explanations regarding both procedures, specifics of tailoring (ie, what and how data will be used for tailoring, for what purposes, such as recommendations, messaging, and user interface designs), advantages, potential benefits, disadvantages, and potential risks with different personalization methods should be provided in the consent forms, facilitating informed and autonomous choices [19]. Given the diversity of characteristics, experiences with different personalization methods, preferences, needs, and concerns, supporting autonomy and agency in choices regarding various personalization methods or not being personalized is essential for ethical and respectful practices [20]. This means that service users can choose between multiple personalization methods, possibly combinations of multiple methods (if available), or choose not to be personalized.

In addition, while there can be multiple personalization methods and multiple scales included for personalization purposes, it is essential to respect service users’ choices in the intended purposes they want to or do not want to be personalized for. This ensures that the data collected are limited and relevant for specific purposes preferred by service users (data minimization), as emphasized in the EU General Data Protection Regulation Article 5(1)(c) [87]. The purpose of personalization (such as for mental health self-care activity recommendation and for reminder or persuasive messages encouraging self-care) of a scale can be specified, allowing service users to make informed decisions on whether they fill in such items.

Service users should also have the right to switch between different personalization methods or switch from being personalized to not being personalized (or vice versa), for example, if they feel uncomfortable or have privacy concerns regarding being personalized in a certain way [19]. Service users may also choose to delete personal data provided or modify the personal records [19], such as the responses to the ultrashort scales, as attributes of some people can change over time. Autonomy in personalization or nonpersonalization choices is critical, given implications for enhancing engagement and psychological well-being, which are key priorities in digital mental health services [20].

Moreover, another potential ethical concern is that algorithmic approaches can risk embedding or amplifying systematic biases present in training data. When personalization is based purely on data-driven correlations, demographic features (eg, age, gender, ethnicity, and education) may inadvertently drive recommendations, leading to discriminatory or inequitable tailoring [88]. For instance, a digital mental health algorithm might offer different content intensities or therapeutic suggestions to subgroups not because of psychological need but due to biased data patterns. A related concern is monophily, the phenomenon where individuals’ attributes can be inferred not only from their own data but also from their network connections or interaction patterns [89]. ML methods trained on these interactions may cause further information divide.

## Limitations and Future Directions

The examples of moderation effects we discussed in this paper are based on a single simple but not multiple samples, and we cannot rule out the possibility of false positives and the possibility of such items having larger regression coefficients due to chance, even though we applied alpha corrections for multiple testing in item-level moderation analyses [90]. In addition, test-retest reliability of the very short forms has not been confirmed. Further studies with additional participant samples, multiple statistical methods, and test-retest reliability tests, perhaps in a digital mental health platform, are needed to examine the validity and reliability as well as replicability, robustness, and generalizability of such (possible) moderation effects before applying broadly in services [33,34]. We also encourage researchers to conduct studies with multiple samples, possibly with multiple statistical methods, before widespread implementation of personalization based on selected items [33,41,91].

While personalization based on 2 to 3 items may seem practically feasible and beneficial on the surface, more studies are needed to test the ecological validity, robustness, replicability, reliability, and generalizability of such methods. Field studies in digital health platforms can be conducted with a personalized group and a nonpersonalized group [92]. In field studies within digital health platforms, perhaps the personalized group of participants may fill in 2 to 3 items that have been shown to be stronger moderators for personalization purposes (eg, participants are assigned to one of the messages based on individual characteristics, short form). Such a personalized (experimental) group can then be compared to the nonpersonalized group (eg, in which participants are assigned to one of the messages randomly). Both objective (eg, number of modules registered, number of modules completed, and duration of usage) and self-report measures (eg, satisfaction and mental health) can be included in digital platforms [9] to assess the efficacy of personalization based on a minimal number of items. We recognize the current empirical base of personalization based on validated ultrashort scales is limited, especially in digital health contexts. The goal of this viewpoint piece is to highlight the gap and to call for future research, but not to systematically synthesize the very scarce literature. We look forward to systematic reviews on this topic when enough studies are conducted in the digital mental health domain.

In addition, future studies may compare the efficacy and effectiveness of personalization based on 2 to 3 items, personalization based on the original scale, personalization based on behavioral data (eg, type of courses registered) tracked in platforms, and personalization based on large language model chatbots. Another issue is that users are probably more likely to complete the minimal number of items than if the number of items is more than 10 [23], but how much more likely? Future studies are needed to empirically test this. Another worthwhile direction is to investigate users’ preferences, attitudes, and concerns regarding being personalized based on a minimal number of items, when compared to being personalized based on a more lengthy scale, tracked behavioral data or being personalized based on large language model chatbots (see the paper by Pieritz et al [20]). It is also possible that people may prefer being personalized based on cognitive, motivational, and decisional characteristics compared to mental health symptoms or diagnosis, as mental health information may be more personally sensitive [19,20]. Future studies can test this speculation. These methods are not mutually exclusive and individual differences data may be captured by a combination of these methods, and personalization choices can be based on a combination or integration of these methods, if considered as acceptable and desirable by individuals.

## Conclusion

To summarize, we propose that personalization based on a minimal number of items of psychological attributes, perhaps through scale shortening and/or through selecting the items that show stronger moderation effects, can be worthwhile. This is also likely more practical and can facilitate personalization for more service users than traditional psychological questionnaires that consist of a larger number of items [23], as more service users may be more willing to fill in a small number of items. Researchers and practitioners may choose the suitable methods (eg, FAs, ML methods, IRT, ACO, and item-level regression analyses) considering strengths, limitations, and feasibility of different methods, depending on factors such as sample size, researchers’ familiarity with methods, and number of factors (also see the papers by Gonzalez [33], Koğar [34,41], and Raborn et al [91]). Our presented 3-tier decision framework may facilitate such choices and implementations. If feasible, the results of 2 or more methods can be considered in selecting the items. While our work focuses on digital mental health, such methods may also be relevant for other digital health domains and we encourage more research on scale shortening in various digital health domains.

While these ideas may seem promising, we call for more studies attempting to investigate diverse user experiences, different user attitudes, efficacy, effectiveness, ecological validity, generalizability, replicability, challenges, disadvantages, and ethical concerns of personalization based on a very small number of items before widespread implementations. Finally, we encourage researchers and practitioners to consider and analyze carefully and considerately regarding the length of scales adopted for personalization purposes in digital mental health contexts. We can aim to increase the likelihood of participants’ filling in personalization-related measures with the potential of further enhancing engagement and reducing participants’ reluctance and discomfort with filling in long questionnaires, while maintaining sufficient validity and reliability with shortened scales and respecting participants’ informed choices of personalization methods, personalization purposes, or nonpersonalization (including opting out or removal of data).

## Supplementary material

10.2196/80662 Multimedia Appendix 1 Supplementary tables of data analyses.

## Abbreviations

ACO: ant colony optimization
ETS: Experiential Thinking Style
FA: factor analysis
GAD-2: Generalized Anxiety Disorder-2
GAD-7: Generalized Anxiety Disorder-7
IRT: item response theory
ML: machine learning
PHQ-2: Patient Health Questionnaire-2
PHQ-4: Patient Health Questionnaire-4
PHQ-9: Patient Health Questionnaire-9
