# Supplementary material for: Using Ultra-Abridged Individual Difference Scales for Personalization in Digital Mental Health to Improve Uptake, Engagement, and Experiences: Three-Tiered Decision Framework for Scale Shortening
Source: J Med Internet Res. 2026 Jun 8;28:e80662. doi: 10.2196/80662 (PMC13247296; doi:10.2196/80662)
Supplement: Multimedia Appendix 1 [file jmir-v28-e80662-s001.docx]

**Supplementary Tables**

The following regression analyses were conducted with RMarkdown [1], through R glm function (family = binomial).

Table S1

*Norris and Epstein [2] Experiential Thinking Style Effect Sizes for Each Item*

| Item Number | Statement | Standardized interaction regression coefficient (95% CI) | Corresponding odds ratio (95% CI) | *P* value |
| --- | --- | --- | --- | --- |
| ETS_1 | I like to rely on my intuitive impressions. | 0.81(0.15–1.5) | 2.26 (1.16 –4.5) | .018 (above adjusted alpha of .015) |
| ETS_2 | I often go by my instincts when deciding on a course of action. | 1.08(0.34-1.83) | 2.96 (1.48 – 6.25) | .003 (below adjusted alpha of .005) |
| ETS_3 | I don’t think it is a good idea to rely on ones intuition for important decisions. (Reversed) | -0.14 (-0.79 – 1.51) | 0.87 (0.45 – 1.66) | .669 |
| ETS_4 | I trust my initial feelings about people. | 0.25(-0.39 – 0.90) | 1.28(0.68 – 2.45) | .448 |
| ETS_5 | I tend to use my heart as a guide for my actions. | 0.90(2.44-1.58) | 2.45(1.28 – 4.85) | .008 (below adjusted alpha of .010) |
| ETS_6 | I enjoy learning by doing something, instead of figuring it out first. | 0.49(-0.15 – 1.14) | 1.63(0.86 – 3.13) | .139 |
| ETS_7 | I can often tell how people feel without them having to say anything. | 0.16(-0.49 – 0.81) | 1.17(0.61 – 2.24) | .632 |
| ETS_8 | I generally don’t depend on my feelings to help me make decisions. (Reversed) | 0.21(-0.44 – 0.86) | 1.23(0.65 – 2.36) | .529 |
| ETS_9 | For me, descriptions of actual people’s experiences are more convincing than discussions about ‘‘facts’’.  I’m not a very spontaneous person. (Reversed) | 0.31(-0.34 – 0.98) | 1.37(0.71 – 2.67) | .350 |
| ETS_10 | I’m not a very spontaneous person. (Reversed) | 0.34(-0.31 – 0.99) | 1.40(0.73 – 2.70) | .307 |

Note: All models used standardized item scores as predictors. Logistic regression models included message condition (narrative vs. research-based evidence) × item interaction terms, adjusted for standardized baseline stress management scores. The score of ETS full form is calculated based on averaging the items after reverse-coding ETS_3, ETS_8, ETS_9, and ETS_10, with the short-form score being the average of ETS_2 and ETS_5. The adjusted alphas for multiple testing are based on Benjamini-Hochberg False Discovery Rate method [3], which is generally more suitable for exploratory tests [4].

The results reported in Tables S1 to S10 are based on an experimental study with narrative messages and research messages, aiming to encourage stress management and identify individual difference moderators. 166 participants were recruited through the Chinese University of Hong Kong (CUHK) subject pool, with undergraduate students in Hong Kong, in 2024.

Table S2

*Hamilton et al. [5] Rational Decision Style Effect Sizes for Each Item*

| Item Number | Statement | Standardized interaction coefficient (95% CI) | Corresponding odds ratio (95% CI) | *P* Value |
| --- | --- | --- | --- | --- |
| DSS_1 | I prefer to gather all the necessary information before committing to a decision. | -0.63(-1.32– 0.14) | 0.53 (0.27– 1.01) | .062 |
| DSS_2 | I thoroughly evaluate decision alternatives before making a final choice. | -0.69(-1.39– -0.035) | 0.50 (0.25 – 0.97) | .044 (above adjusted alpha of .025) |
| DSS_3 | In decision making, I take time to contemplate the pros/cons or risks/benefits of a situation. | -0.25(-0.96 – 0.42) | 0.78 (0.38 – 1.53) | .469 |
| DSS_4 | Investigating the facts is an important part of my decision making process. | -0.59(-1.31 – 0.11) | 0.55 (0.27 – 1.11) | .099 |
| DSS_5 | I weigh a number of different factors when making decisions. | -0.22(-0.88 – 0.43) | 0.80 (0.41 – 1.53) | .507 |

Note: All models used standardized item scores as predictors. Logistic regression models included message condition (narrative vs. research-based evidence) × item interaction terms, adjusted for standardized baseline stress management scores. The score of DSS Rational subscale should be calculated based on averaging the above five items.

N = 166 participants recruited through the CUHK subject pool with undergraduate students in Hong Kong.

The full Rational Decision Style subscale moderated the effect of message condition on breathing-exercise decisions, *b* = –0.77, 95% CI [–1.48, –0.06], *p* = .035, *OR* = 0.46, 95% CI [0.23, 0.95]. The two-item short version (Items 1 and 2, with the score based on average of these two items) showed a similar pattern, *b* = –0.78, 95% CI [–1.47, –0.10], *p* = .025, *OR* = 0.46, 95% CI [0.23, 0.91]. Internal consistency for the full version was α = .78 (inter-item correlation = 0.42), and the short version yielded a Spearman-Brown coefficient of .69 (inter-item correlation = 0.52). The two versions were strongly correlated, *r(*164) = .85, 95% CI [.80, .89], *p* < .001.

Table S3

*Hamilton et al. [5] Intuitive Decision Style Effect Sizes for Each Item*

| Item Number | Statement | Standardized interaction coefficient(95% CI) | Corresponding odds ratio(95% CI) | *P* Value |
| --- | --- | --- | --- | --- |
| DSS_6 | When making decisions, I rely mainly on my gut feelings. | 1.09(0.41–1.81) | 2.97(1.51–6.11) | .002 (below adjusted alpha of .005) |
| DSS_7 | My initial hunch about decisions is generally what I follow. | 0.80(0.14–1.49) | 2.23(1.15–4.44) | .020 (above adjusted alpha of .015) |
| DSS_8 | I make decisions based on intuition. | 0.89(0.24–1.59) | 2.45(1.27–4.88) | .009 (below adjusted alpha of .010) |
| DSS_9 | I rely on my first impressions when making decisions. | 0.50(-0.16–1.17) | 1.64(0.85–3.23) | .144 |
| DSS_10 | I weigh feelings more than analysis in making decisions. | 0.72(-0.064–1.39) | 2.04(1.07–4.02) | .034 (above adjusted alpha of .020) |

Note: All models used standardized item scores as predictors. Logistic regression models included message condition (narrative vs. research-based evidence) × item interaction terms, adjusted for standardized baseline stress management scores. The score of DSS Intuitive scale should be calculated by averaging the above 5 items.

*N* = 166 participants recruited through the CUHK subject pool.

The full Intuitive Decision Style subscale significantly moderated the effect of message condition on breathing-exercise decisions, *b* = 0.99, 95% CI [0.29, 1.69], *p* = .005, *OR* = 2.70, 95% CI [1.34, 5.45]. The two-item short version (Items 6 and 8, with the score calculated by averaging these two items) showed a similar pattern, b = 1.04, 95% CI [0.35, 1.73], *p* = .003, *OR* = 2.82, 95% CI [1.42, 5.62]. Internal consistency for the full version was α = .86 (inter-item correlation = 0.54), and the short version yielded a Spearman-Brown coefficient of .88 (inter-item correlation = 0.78). The two versions were strongly correlated, r(164) = .91, 95% CI [.88, .93], *p* < .001.

Table S4

*Simple Slope Analyses*

| Moderator | Level | OR [95% CI] | p-value |
| --- | --- | --- | --- |
| Rational Decision-Making Style(Full) | Low (–1 SD ≈ 3.56) | *OR* = 2.32 [0.91, 5.90] | *p* = .077 |
|  | Mean (≈ 4.05) | *OR* = 1.08 [0.56, 2.07] | *p* = .824 |
|  | High (+1 SD ≈ 4.55) | *OR* = 0.50 [0.18, 1.35] | *p* = .172 |
| Rational Decision-Making Style(Short) | Low (–1 SD ≈ 3.36) | *OR* = 2.50 [0.97, 6.45] | *p* = .059 |
|  | Mean (≈ 4.00) | *OR* = 1.14 [0.60, 2.17] | *p* = .691 |
|  | High (+1 SD ≈ 4.64) | *OR* = 0.52 [0.20, 1.32] | *p* = .170 |
| Intuitive Decision-Making Style(Full) | Low (–1 SD ≈ 2.22) | *OR* = 0.40 [0.16, 1.07] | *p* = .060 |
|  | Mean (≈ 2.99) | *OR* = 1.11 [0.57, 2.14] | *p* = .758 |
|  | High (+1 SD ≈ 3.75) | *OR* = 2.74 [1.14, 7.87] | *p* = .026 |
| Intuitive Decision-Making Style(short) | Low (–1 SD ≈ 2.05) | *OR* = 0.40 [0.16, 1.03] | *p* = .058 |
|  | Mean (≈ 2.97) | *OR* = 1.13 [0.58, 2.19] | *p* = .717 |
|  | High (+1 SD ≈ 3.88) | *OR* = 3.19 [1.21, 8.36] | *p* = .019 |
| Experiential–Intuitive Thinking Style(Full) | Low (–1 SD ≈ 2.73) | *OR* = 0.46 [0.18, 1.17] | *p* = .102 |
|  | Mean (≈ 3.21) | *OR* = 1.15 [0.60, 2.19] | *p* = .683 |
|  | High (+1 SD ≈ 3.70) | *OR* = 2.85 [1.10, 7.41] | *p* = .031 |
| Experiential–Intuitive Thinking Style(Short) | Low (–1 SD ≈ 2.37) | *OR* = 0.35 [0.14, 1.92] | *p* = .034 |
|  | Mean (≈ 3.17) | *OR* = 1.13 [0.58, 2.21] | *p* = .715 |
|  | High (+1 SD ≈ 3.97) | *OR* = 3.62 [1.37, 9.58] | *p* = .010 |

Table S5

*Correlations with confidence intervals between variables measured in Zhao et al. [6]*

| Variable | 1 | 2 | 3 | 4 | 5 | 6 | 7 | 8 |
| --- | --- | --- | --- | --- | --- | --- | --- | --- |
|  |  |  |  |  |  |  |  |  |
| 1. ETS _full |  |  |  |  |  |  |  |  |
|  |  |  |  |  |  |  |  |  |
| 2. ETS _short | .84** |  |  |  |  |  |  |  |
|  | [.79, .88] |  |  |  |  |  |  |  |
|  |  |  |  |  |  |  |  |  |
| 3. DSS _rational_full | -.29** | -.29** |  |  |  |  |  |  |
|  | [-.42, -.15] | [-.42, -.14] |  |  |  |  |  |  |
|  |  |  |  |  |  |  |  |  |
| 4. DSS _intuitive_full | .68** | .74** | -.21** |  |  |  |  |  |
|  | [.58, .75] | [.66, .80] | [-.35, -.06] |  |  |  |  |  |
|  |  |  |  |  |  |  |  |  |
| 5. DSS _rational_short | -.25** | -.25** | .85** | -.16* |  |  |  |  |
|  | [-.39, -.10] | [-.39, -.10] | [.80, .89] | [-.30, -.01] |  |  |  |  |
|  |  |  |  |  |  |  |  |  |
| 6. DSS _intuitive_short | .61** | .68** | -.25** | .91** | -.17* |  |  |  |
|  | [.50, .69] | [.59, .76] | [-.39, -.10] | [.88, .93] | [-.31, -.01] |  |  |  |
|  |  |  |  |  |  |  |  |  |
| 7. NFC | -.01 | -.06 | .06 | -.16* | .02 | -.10 |  |  |
|  | [-.17, .14] | [-.21, .09] | [-.09, .21] | [-.30, -.00] | [-.13, .17] | [-.24, .06] |  |  |
|  |  |  |  |  |  |  |  |  |
|  |  |  |  |  |  |  |  |  |
| 9. subjective_numeracy | -.06 | -.20** | .25** | -.13 | .19* | -.12 | .25** |  |
|  | [-.21, .09] | [-.34, -.05] | [.10, .39] | [-.28, .02] | [.03, .33] | [-.27, .03] | [.11, .39] |  |
|  |  |  |  |  |  |  |  |  |

*Note.* This table is created and adapted based on apaTables R package [7]. The square bracekets refer to the 95% confidence intervals of each correlation.* indicates *p* < .05. ** indicates *p* < .01. NFC refers to the need for cognition short form by Lins de Holanda Coelho et al. [8] whereas subjective numeracy refers to the scale by Fagerlin et al. [9].

Table S6

*Inter-Item Correlations for experiential thinking style full form by Norris and Epstein [2]*

| Variable | 1 | 2 | 3 | 4 | 5 | 6 | 7 | 8 | 9 |
| --- | --- | --- | --- | --- | --- | --- | --- | --- | --- |
|  |  |  |  |  |  |  |  |  |  |
| 1. ets_1 |  |  |  |  |  |  |  |  |  |
|  |  |  |  |  |  |  |  |  |  |
| 2. ets_2 | .74** |  |  |  |  |  |  |  |  |
|  | [.66, .80] |  |  |  |  |  |  |  |  |
|  |  |  |  |  |  |  |  |  |  |
| 3. ets_3r | .36** | .38** |  |  |  |  |  |  |  |
|  | [.22, .48] | [.24, .51] |  |  |  |  |  |  |  |
|  |  |  |  |  |  |  |  |  |  |
| 4. ets_4 | .40** | .29** | .14 |  |  |  |  |  |  |
|  | [.26, .52] | [.15, .43] | [-.01, .29] |  |  |  |  |  |  |
|  |  |  |  |  |  |  |  |  |  |
| 5. ets_5 | .47** | .46** | .19* | .39** |  |  |  |  |  |
|  | [.35, .58] | [.33, .57] | [.04, .33] | [.25, .51] |  |  |  |  |  |
|  |  |  |  |  |  |  |  |  |  |
| 6. ets_6 | .24** | .28** | -.01 | .13 | .27** |  |  |  |  |
|  | [.09, .38] | [.13, .41] | [-.16, .14] | [-.02, .27] | [.12, .41] |  |  |  |  |
|  |  |  |  |  |  |  |  |  |  |
| 7. ets_7 | .16* | .07 | .10 | .25** | .27** | .10 |  |  |  |
|  | [.00, .30] | [-.08, .22] | [-.05, .25] | [.11, .39] | [.13, .41] | [-.05, .25] |  |  |  |
|  |  |  |  |  |  |  |  |  |  |
| 8. ets_8r | .24** | .25** | .15 | .04 | .30** | .04 | .05 |  |  |
|  | [.10, .38] | [.11, .39] | [-.00, .29] | [-.11, .19] | [.15, .43] | [-.11, .19] | [-.10, .20] |  |  |
|  |  |  |  |  |  |  |  |  |  |
| 9. ets_10 | -.06 | .01 | -.06 | .08 | .06 | .01 | .02 | -.17* |  |
|  | [-.21, .10] | [-.14, .17] | [-.21, .09] | [-.07, .23] | [-.10, .21] | [-.14, .16] | [-.14, .17] | [-.32, -.02] |  |
|  |  |  |  |  |  |  |  |  |  |
| 10. ets_11r | .17* | .19* | .12 | -.02 | .13 | .15 | -.00 | .23** | -.09 |
|  | [.02, .31] | [.04, .33] | [-.04, .26] | [-.17, .13] | [-.03, .27] | [-.00, .29] | [-.15, .15] | [.08, .37] | [-.24, .06] |
|  |  |  |  |  |  |  |  |  |  |

Table S7

*Inter-Item correlations for Rational Decisional-making Scale by Hamilton et al. [5]*

| Variable | 1 | 2 | 3 | 4 |
| --- | --- | --- | --- | --- |
|  |  |  |  |  |
| 1. dss_1 |  |  |  |  |
|  |  |  |  |  |
| 2. dss_2 | .52** |  |  |  |
|  | [.40, .63] |  |  |  |
|  |  |  |  |  |
| 3. dss_3 | .31** | .48** |  |  |
|  | [.17, .44] | [.36, .59] |  |  |
|  |  |  |  |  |
| 4. dss_4 | .36** | .36** | .45** |  |
|  | [.22, .49] | [.22, .48] | [.32, .56] |  |
|  |  |  |  |  |
| 5. dss_5 | .31** | .42** | .52** | .48** |
|  | [.16, .44] | [.29, .54] | [.40, .62] | [.35, .59] |
|  |  |  |  |  |

Table S8

*Inter-Item correlations for Intuitive Decisional-making Scale by Hamilton et al. [5]*

| Variable | 1 | 2 | 3 | 4 |
| --- | --- | --- | --- | --- |
|  |  |  |  |  |
| 1. dss_6 |  |  |  |  |
|  |  |  |  |  |
| 2. dss_7 | .57** |  |  |  |
|  | [.46, .66] |  |  |  |
|  |  |  |  |  |
| 3. dss_8 | .78** | .61** |  |  |
|  | [.72, .84] | [.50, .69] |  |  |
|  |  |  |  |  |
| 4. dss_9 | .52** | .52** | .57** |  |
|  | [.40, .63] | [.40, .63] | [.46, .66] |  |
|  |  |  |  |  |
| 5. dss_10 | .51** | .38** | .51** | .47** |
|  | [.38, .61] | [.24, .50] | [.38, .61] | [.34, .58] |
|  |  |  |  |  |

Table S9

*Exploratory Factor Analysis for Norris and Epstein [2] Experiential Thinking Style - Rotated Factor Matrix (ETS), with Parallel Analysis*

|  | Factor 1 | Factor 2 | Factor 3 |
| --- | --- | --- | --- |
| ETS_2 | .910 / .922 |  |  |
| ETS_1 | .751 / .771 |  |  |
| ETS_3 | / | / | / |
| ETS_6 | / | / | / |
| ETS_5 | / | .605 / .713 | / |
| ETS_4 | / | .505 / .478 | / |
| ETS_7 | / | .485 / .508 | / |
| ETS_8 | / | / | .765 / .611 |
| ETS_10 | / | / | / |
| ETS_9 | / | / | / |

*Note.* Extraction Methods: Maximum Likelihood (left) / Minimum Residuals (right); Rotation Methods: Varimax with Kaiser Normalization (left) / Oblimin (right); Coefficients below 0.4 were suppressed. We conducted the analyses with SPSS [10] and Jamovi [11].

Table S10

*Exploratory Factor Analysis for Hamilton et al. [5] Decision Style - Rotated Factor Matrix (ETS)*

| Item Number | Factor 1 | Factor 2 |
| --- | --- | --- |
| DSS_8 | .881 / .870 | / |
| DSS_6 | .834 / .819 | / |
| DSS_7 | .695 / .721 | / |
| DSS_9 | .653 / .693 | / |
| DSS_10 | .574 / .593 | / |
| DSS_3 | / | .736 / .720 |
| DSS_5 | / | .709 / .700 |
| DSS_2 | / | .673 / .706 |
| DSS_4 | / | .586 / .571 |
| DSS_1 | / | .491 / .505 |

For Rational Decision Style [5], since item 2 is ranked third/second in the EFA factor loadings and ranked first in the item-level regression coefficient, while item 4 is ranked fourth in the EFA factor loadings with a satisfactory value and ranked third in the regression coefficient, we recommend choosing Item 2 and Item 4 for rational decision style in the context of narrative vs research evidence/statistical message framing. Despite higher factor loadings of item 3 and item 5, considering the small regression coefficients, we believe choosing these items are less appropriate for personalization.

For Intuitive Decision Style [5], we suggest choosing Item 8 and Item 6 as they have the highest regression coefficients and factor loadings.

For further simplification, since Intuitive Decision Style has a stronger moderation effect for narrative versus research evidence messaging, choosing Item 8 and Item 6 may be sufficient without including Item 2 and Item 4 from rational style subscale, if such results are replicated in different sample(s) and in the field. Further field studies in digital health platform(s) can test if simply including Item 8 and Item 6 can facilitate personalization for narrative versus research messages effectively.

**Supplementary References**

1. Xie Y, Allaire JJ, Grolemund G. R Markdown: The definitive guide. Chapman and Hall/CRC; 2018.
2. Norris P, Epstein S. An experiential thinking style: its facets and relations with objective and subjective criterion measures. J Pers. 2011;79(5):1043-1080.
3. Benjamini Y, Hochberg Y. Controlling the false discovery rate: a practical and powerful approach to multiple testing. J R Stat Soc Series B Methodol. 1995;57(1):289-300.
4. Menyhart O, Weltz B, Győrffy B. MultipleTesting.com: A tool for life science researchers for multiple hypothesis testing correction. PLoS One. 2021;16(6):e0245824.
5. Hamilton K, Shih SI, Mohammed S. The development and validation of the rational and intuitive decision styles scale. J Pers Assess. 2016;98(5):523-535.
6. Zhao H, Yeung SK, Mak WWS. Narrative versus research‑based messages in encouraging stress management: decision style and experiential thinking style as moderators. Preprint. 2025. Available from: <https://www.researchgate.net/publication/397179578_Narrative_versus_Research-based_Messages_in_Encouraging_Stress_Management_-_Decision_Style_and_Experiential_Thinking_Style_as_Moderators>
7. Stanley DJ, Spence JR. Reproducible tables in psychology using the apaTables package. Adv Methods Pract Psychol Sci. 2018;1(3):415-31.
8. Lins de Holanda Coelho G, Hanel PH, Wolf LJ. The very efficient assessment of need for cognition: Developing a six-item version. Assessment. 2020;27(8):1870-85.
9. Fagerlin A, Zikmund-Fisher BJ, Ubel PA, Jankovic A, Derry HA, Smith DM. Measuring numeracy without a math test: development of the Subjective Numeracy Scale. Medical Decision Making. 2007 Sep;27(5):672-80.
10. George D, Mallery P. IBM SPSS statistics 26 step by step: A simple guide and reference. Routledge; 2019.
11. Şahin M, Aybek E. Jamovi: an easy to use statistical software for the social scientists. Int J Assess Tools Educ. 2019;6(4):670-692.
